# Pilot Study on Satisfaction in Children and Adolescents after a Comprehensive Educational Program on Healthy Habits

**DOI:** 10.3390/nu15051161

**Published:** 2023-02-25

**Authors:** Noelia Belando-Pedreño, Marta Eulalia Blanco-García, José L. Chamorro, Carlos García-Martí

**Affiliations:** 1 Faculty of Sports Sciences, Universidad Europea de Madrid, 28670 Madrid, Spain; 2 Hum-878 Research Team, Health Research Centre, Department of Psychology, University of Almería, 04120 Almeria, Spain

**Keywords:** educational innovation, Mediterranean diet, healthy lifestyles, emotional skills, social skills, schoolchildren

## Abstract

Prospective research in the area of Education Sciences and Physical-Sports Education agree on the need to design and implement educational programs that promote emotional competencies (ECs), interpersonal competencies (ICs), an adequate level of healthy physical activity (NAFS) and a good adherence to the Mediterranean diet (ADM). The main objective of the study is to design an intervention program in intra- and interpersonal competencies together with nutritional education and corporality called “MotivACTION”. The sample consisted of 80 primary schoolchildren aged 8 to 14 years (M = 12.70; SD = 2.76) (37 girls and 43 boys) from two schools in the Community of Madrid. An ad-hoc questionnaire was created to assess the participant’s perception of the usefulness of the “MotivACTION” educational experience. The program “MotivACTION: Feed your SuperACTION” is designed and implemented based on the development of a workshop organized through the Universidad Europea de Madrid. As the main preliminary results of the pilot study, the schoolchildren who experienced the “MotivACTION” workshop showed high satisfaction with the educational program. They were able to create a healthy menu with the frog chef. They also felt better and happier at the end of it, and they enjoyed practicing physical activity moving to the rhythm of the music while doing mathematical calculations.

## 1. Introduction

Promoting healthy habits in children and adolescents (e.g., nutrition, physical activity, mental health, etc.,) should be a priority for institutions, researchers, and education professionals. Educational programs will have a greater or lesser impact based on participant satisfaction. Furthermore, creating educational programs based on motivational principles and offering emotional tools to cope with healthy habits changes could increase satisfaction and, thus, change behaviors. This study presents the evaluation of the “MotivACTION”, an educational program based on motivational principles and aimed at children and adolescents, founded in emotional education, personal and social values education, nutritional education, and promoting more active lifestyle habits through the proper holistic development of the young.

### 1.1. Theoretical Background

The design of the “MotivACTION program” is based on the postulates of various theories that explain human behavior such as the Self-Determination Theory (SDT) [1,2], the Theory of Planned Behavior (TPB) [3], and the Theory of Emotional Intelligence (TEI) [4]. These theoretical paradigms provide keys to incorporate motivational processes in a learning process where students go beyond memorizing knowledge and learn to develop skills such as “knowing how to be” and “knowing how to do” [5].

SDT [1,2] establishes different motivation types according to whether their origin is internal or external to the individual. From the perspective of SDT, students can experience different types of motivation depending on the level of self-determination (intrinsic motivation, extrinsic motivation and amotivation). In addition, this theory posits that people become more committed to a particular activity if their needs for autonomy, competence, and social relatedness are satisfied. When the pedagogical designs implemented by teachers/educators/facilitators satisfy these basic psychological needs, it is more likely that students will have a more self-determined degree of motivation to participate in learning tasks [6,7] and exhibit more active behaviors at the physical level [8] such as personal care and better levels of daily physical activity and, at the educational level, more proactive and social behaviors toward learning [6,9]. In this sense, the “MotivACTION program” consists of activities organized in small and large groups with a guided discovery methodology, which encourages more self-determined motivation and satisfaction of the basic psychological needs to promote the commitment of the participants.

Furthermore, according to the TPB [3], the intention to do something (to have a certain behavior) is, in turn, influenced by three basic determinants: two of a personal nature (such as motivation) and another that reflects social influence. One of them is the attitude toward the behavior, that is, the positive or negative evaluation a person makes just before “taking action” (manifesting the behavior). The second of these, of a social nature, refers to the subjective norm, that is, the person’s perception of the pressures from peers or other people in the context (negative comments, or positive feedback) that are exerted on her to perform the expected behavior. And the third is perceived behavioral control, that is, to what extent the person feels in control of executing the behavior. Perceived control can directly predict behavior depending on whether it is under voluntary control and whether there are discrepancies between the control the person believes they have and the control they have. So, if the students/participants perceive themselves with the real and necessary ability to act and also feel motivated toward it, they could act. Therefore, from the “MotivACTION program”, situations are worked on in which participants have to put in their effort, that is, to show a proactive attitude toward the desired behavior. In addition, they are encouraged with positive, prescriptive, and interrogative feedback (subjective norm) and have to face challenges/tasks which they feel they can do and enjoy while resolving the situation (perception of behavior control). In this sense, the performance of physical exercise as a healthy behavior integrated into a learning context contributes to the perception of control [10] and better predicts behavior, compared to attitude and subjective norm, the latter being the one that predicts the worst [10].

Moreover, according to Goleman [4], emotional intelligence is understood as the set of socio-emotional competencies related to success in work or any area of personal development. García-Fernández and Giménez-Más [11] proposed a model of emotional intelligence that encompasses the study of both internal or endogenous aspects (particular traits of the individual, which can be innate or acquired through learning or knowledge) and external or exogenous aspects (which are behaviors based on adaptability to the environment). In MotivACTION, practices focus on endogenous factors such as responsibility (capacity to understand the consequences of actions in the future), common sense, and the ability to learn and even unlearn, which does not help us progress toward personal and social goals such as adopting healthy habits. The most encouraged exogenous factors are the ability to adapt to a changing environment (very important in the last two years of the COVID-19 pandemic), empathy (predisposition to understand what others are going through and support them in that process), and the ability to communicate assertively (using kind and positive language toward ourselves and others). These are all strategies that help to face the challenges of adopting healthy habits.

### 1.2. Nutrition Education as a Strategy for Promoting Healthy Habits in Children

Nutrition education (NE) refers to all educational activities that involve students and the entire educational community (socializing agents: teachers, educational institutions, families) through direct education in the classroom and the transfer of this education to the context of personal, family, and social development. The aim is to motivate students to adopt healthy eating behavior and lifestyle choices in general. Thus, the acquisition of integral health and the promotion of healthy habits in educational environments has become one of the most important challenges, mainly due to the fact that childhood obesity has become an epidemic pathology worldwide [12]. In 2021, the WHO [13] reported alarming figures for overweight and obesity in young children: 39 million children under 5 years of age were overweight or obese in 2020; more than 340 million children and adolescents aged 5–19 years were overweight or obese in 2019. To reduce these figures and the future consequences on physical health (development of chronic non-communicable diseases such as obesity, diabetes, hypercholesterolemia), emotional health, and the quality of social relations of schoolchildren and adolescents, educational intervention strategies are needed at the government level aimed at promoting healthy habits through nutritional education and an active lifestyle [14,15]. In this sense, there are various strategies in the field of nutritional health promoted by world bodies such as the WHO (2004) [16] with the Global Strategy on Diet, Physical Activity and Health, with objectives such as: Reducing the risk factors for chronic diseases derived from unhealthy diets and sedentary lifestyles through public health actions; increasing awareness and understanding of the influences of diet and physical activity on health and the positive impact of preventive interventions. In the same line of action, the NAOS Strategy (Nutrition, Physical Activity and Obesity Prevention) of the Ministry of Consumer Affairs (Spanish government) aims to reduce the prevalence of obesity by promoting healthy eating and physical activity. There are other relevant initiatives in Spain, coordinated by the Ministry of Consumer Affairs and AESAN (acronym in Spanish for the Spanish Agency of Food Safety and Nutrition) through the “observatory of nutrition and the study of obesity”, which propose lines of work to promote healthy and sustainable eating in schools and other public centers (i.e., campaign Put more heroes on your plate, and fill your life with superpowers, 2022 [17]).

In terms of NE research, several literature review studies and meta-analyses show the positive effects of multicomponent nutrition education programs (eating behavior strategies, motivational strategies, meal planning strategies, etc.,) [18,19,20,21]. It seems that the most successful intervention methodologies include multiple strategies and an “active” approach (methodologies capable of producing effective changes in eating habits and not only disseminating nutritional information) [22]. Furthermore, most of the intervention programs carried out in recent years to counteract childhood obesity have been those carried out in schoolchildren aged 6–12 years, which addressed practical contents such as: (a) Focusing actions on eating behavior, rather than a knowledge-based approach alone; (b) focusing on individual and environmental behaviors related to diet and physical activity level; (c) educational and practical component programs (video lessons or video labs, implementation of content through meaningful activities such as gamified challenges or cooking workshops) [19,23]; (d) didactic content promoting healthy nutrition, physical activity, guiding parents on how to do to improve their relationship with their children [24], among others.

“MotivACTION” is an innovative educational approach that integrates knowledge and practical activities on emotional education, physical education, and education in proper eating behavior. Healthy eating and regular physical exercise are highlighted as necessary actions to maintain or improve well-being and, in turn, promote human health [25,26]. From a nutritional perspective, a healthy diet provides all the types and amounts of foods and nutrients that the body needs to function properly and prevent the development of diseases due to nutritional excess or deficiency. For this to occur, several factors are necessary, including: (a) optimal food security, i.e., that food (in quantity and variety) is available and affordable in the community; (b) that available and affordable food is safe and free from contaminants and substances that can harm the body once ingested [27]; (c) that the individual consumes an adequate variety and quantity of foods, tailored to their nutritional needs; (d) that the process of nutrition (the involuntary process by which nutrients are digested and absorbed) is functioning properly [26]. All these factors influence, in one way or another, preventing or combating the two extremes of malnutrition that coexist today. In this sense, one of the main objectives of “MotivACTION” is for children and adolescents to identify which emotions and day-to-day situations influence their behavior and how this impacts their daily physical activity level and eating behavior in and out of the classroom.

### 1.3. Aim

The main objective of this study is to test the satisfaction of children and adolescents with the design and implementation of a “pilot” educational program called “MotivACTION, feed your superACTION” as an integrative training strategy for proper emotional education, nutritional education, and promotion of active lifestyle habits in children and adolescents.

### 1.4. Hypothesis

After the acute implementation of the MotivACTION educational program, participants will show a high level of satisfaction and achievement. Furthermore, it is hypothesized that the participants will perceive the importance of transferring the learning acquired in the program to everyday life (classroom situations, family life).

## 2. Materials and Methods

### 2.1. Design

The research design corresponds to a protocol or methodological development design of an educational program. A pilot study of an Action Research type (research is conducted at the same time as intervention) was carried out in an educational and social context [28] for the implementation of the program.

### 2.2. Participants

The sample consisted of 80 primary and secondary school students aged 8 to 14 years old (M = 12.70; SD = 2.76) (37 girls and 43 boys) from two educational centers and teenagers attending an academic support center in the Community of Madrid (Table 1).

Among the criteria for the selection of participants, the variables of age, sex, educational stage, and socio-economic level. The sampling technique applied was non-probabilistic by convenience [29]. The educational centers, parents/guardians of the students and teenagers gave approval for the minors’ participation in the scientific-technical activity and informed consents were signed.

This study has been designed and implemented considering all the bioethical principles established by the Belmont Report [30] and the Declaration of Helsinki [31]: principles of autonomy, beneficence, justice, and non-maleficence.

### 2.3. Instruments

An ad-hoc questionnaire was created to evaluate the participant’s perception of the educational experience “MotivACTION, feed your superACTION”. This instrument combines the collection of quantitative data through 9 Likert-type questions (e.g., How much do you feel that it has helped you become more aware of the importance of your thoughts and emotions?) rated from 0 (worst or least liked) to 10 (best or most liked). In addition, it included an open-ended question, number 10, to find out participants’ perceptions of the transferability of the workshop in different contexts of their lives: What have you learned in the workshop that you find useful in your daily life in school/institute, in your relationship with your classmates, and in your relationship with your family?

This questionnaire was created to evaluate different participants’ perceptions of the educational program. Thus, the questionnaire is made up of a set of single items that refers to different dimensions or unifactorial constructs. According to Angulo-Brunet et al. (2020) [32], it is not suitable to test the psychometric properties of single-item measures through measurement models and internal consistency reliability coefficients. Instead, they propose to test the evidence of the validity of the single-item measures through item content validity and the response process. In our study, evidence on item content validity was tested by a panel of two experts that evaluated if the contents of every single item reflect what the researchers wanted to measure. Validity evidence related to the response process was first obtained through a cognitive interview in the development phase and second, relying on participants’ comments during data collection [32].

To recruit the sample, the schools were contacted by telephone, the head of studies was contacted, and the objective of the action research was communicated to them as part of a scientific transfer and dissemination activity organized by the Universidad Europea de Madrid. Once the approval of the heads of the schools was obtained, a meeting was organized with the teachers in order to count on their collaboration in the development of the “MotivACTION, feed your superACTION” workshop. Likewise, the relatives of the participants were informed of the objective of the study and the type of tests (questionnaire) to be implemented for data collection, and the anonymity of the responses was preserved.

### 2.4. Procedure

The educational program “MotivACTION, feed your superACTION” was carried out under the workshop format of the same name on the occasion of the “International Day of Women and Girls in Science” and as part of other scientific dissemination activities in the Community of Madrid linked to the “European Project Madrid+d”. These dissemination events have been organized and approved by the Universidad Europea de Madrid for the 2021/2022 academic year. The program was coordinated by a maximum of two university teachers with doctorates in Physical Activity and Sports Sciences and Biology, respectively, with knowledge in Health Sciences (emotional education, motor behavior and nutritional education). In the case of workshops for small groups (6–8 participants), they would be facilitated by one teacher.

### 2.5. Design and Application of the Educational Program

The educational program consisted of two educational workshops called “MotivACTION, feed your superACTION” structured along eight thematic lines (Table 2) related to emotional skills work, awareness of cognitions (thoughts), nutritional education, and physical education (exercise techniques) related to other curricular subjects. The maximum duration of each workshop was 1 h and 30 min.

The “MotivACTION” program was designed based on the scientific evidence on the psychological construct “intrinsic motivation” that is analyzed and developed in Reeve [33] (see chapter 5, page 83), as well as the internal and external motives that determine a person’s behavior. The “MotivaACTION” program is also based on the postulates of various theories that explain human behavior: SDT (it was applied in activities of recognition of different motivational states that students have in their daily lives, or in activities, whose objective was to know the motives of sports practice); TPB (it was carried out in physical activities developed during the workshop in which participants showed a proactive attitude towards behavior, in which they were incentivized through positive, prescriptive and interrogative feedback (subjective norm) and faced physical challenges in which they had to make decisions among the group of peers (perception of behavioral control). The postulates of emotional intelligence (Theory Emotional Intelligence, TEI) were used to work on Dr. Hitzig’s emotional literacy (reflection and debate on what kind of emotions and associated attitudes occur in different situations in the classroom, at playtime, in the canteen, in sports practice with friends, at home in the relationship with the family, among others).

The nutritional part of “MOTIVACTION” is based on the recommendations of the FAO [34] and the Spanish Food Safety and Nutrition Agency (AESAN) [17], with a didactic, fun (using humor to promote positive emotions) role-playing situations, group reflections practical approach to daily life. The topics that the didactic workshop on nutrition covers are: (a) Integrating and enjoying a wide variety of foods and dividing consumption into five to six small meals/day; (b) how to make a nutritious and simple breakfast every day; (c) what types of cereals exist, how and when to consume them; (d) what micronutrients are and how to get them through daily portions of fruits and vegetables; (e) healthy and “attractive” alternatives to “fast food” to reduce high-fat and added-sugar foods; (f) who “lives” in the gut walls: the microbiota and its care; (g) why and when to hydrate throughout the day and when performing a certain amount and intensity of physical activity; (h) reviewing the importance of body composition beyond total weight and what daily actions to take to stay active.

### 2.6. Data Analysis

A descriptive analysis (mean and standard deviation) was performed on the Likert-type responses from 1 to 9 of the ad-hoc questionnaire. Regarding qualitative analysis, a content analysis was carried out through the transcription of the responses given by each participant to question number 10. Excel software in Excel Book (.xlsx) format for Mac was used for the descriptive analysis.

Regarding qualitative analysis, to find common themes and patterns in the responses to item 10, a content analysis was performed with a deductive/inductive approach [35]. Then, the cites that best reflected the personal experiences of the participants in relation to the research objective were identified and coded, creating different categories with representative meanings [36]. For greater reliability, an internal consistency analysis was carried out in which the four authors participated in order to increase their precision standard and corroborate the consistency of the results [35].

## 3. Results

### 3.1. Quantitative Results

Table 3 shows the mean and standard deviation of all items of the questionnaire. The findings show that most items in the questionnaire received high mean scores from the participants. It is important to note that participants found the program useful to improve their way of thinking, their emotions, and their fitness level. The items most highly rated by the participants (M > 9.00) were: item (8) “Rate the activity in terms of organization”; item (7) “Rate the activity in terms of format”; item (3) “How much do you feel that becoming more aware of the importance of your thoughts and emotions has contributed to you?” Item (1) “How useful is it for you to work on your way of thinking, your emotions, and your fitness level?” The commitment to continue participating in the program and to recommend to other peers had also high punctuation. Overall, the general satisfaction with the program was 8.76.

### 3.2. Qualitative Results

The qualitative results show the perception about the educational program “MotivACTION, feed your superACTION”. These results refer to the students’ answers to the semi-open question: What did you learn in the workshop that is useful for your day-to-day life at school, in your relationship with your classmates, and in your relationship with your family? (Item 10 ad-hod questionnaire). Three categories for each of the contexts emerged from the content analyses (Table 4). In the first context (day-to-day life at school), the educational program influenced the attention of the participants to what the teacher is saying, to pass a better time in class and to express what they feel in stressful situations. In the second context (relationships with their families), the participants improve their skills to tell their parents when they feel bad, make healthier plates for lunch and dinner and being more active with their family. In the first context (relationships with their classmates), the educational program improves the quality of time that they spend with their classmates and to be more active with them.

## 4. Discussion

This study aimed to test the satisfaction of children and adolescents with the design and implementation of a “pilot” educational program called “MotivACTION, feed your superACTION” as an integrative training strategy for proper emotional education, nutritional education, and promotion of active lifestyle habits in children and adolescents. Eating practices and young students’ perception of healthy lifestyle habits (eating, physical exercise, satisfactory social relations, perceived well-being, etc.,) seem to be determined also by external factors, such as the food, cultural, school, and social environment and socio-economic status [37,38,39]. In this sense, the quantitative results of the ad-hod questionnaire showed the importance given by schoolchildren to the organizational aspect, to the format in which the information is presented in the “MotivACTION” workshop (activities that apply to daily life, with group dynamics, using humor, peer interaction, music as an element to motivate the rhythmic movement of the different body segments, etc.,). In the same line of study, a recent study based on different nutritional strategies developed in Indonesian adolescents [40] showed consistent results on food awareness and healthier lifestyles when programs with an appropriate organizational structure, facilitated by teachers/school staff, focused on behavior change related to healthy nutrition and physical activity, are carried out as part of a package of interventions for the improvement of the overall health of young students [41,42].

Concerning the items being more aware of the importance of your thoughts and emotions and how useful they think it is for them to work on their thinking, emotions, and physical fitness (3 and 1, respectively), there is a fundamental aspect to pay special attention to in the school and adolescent population: optimal mental health understood as a state of well-being in which a person is aware of his or her capabilities, being able to cope with normal day-to-day stresses, carrying out academic tasks productively, and being able to participate in his or her community (e.g., in the educational environment) in an active manner [43]. In this regard, the WHO adopted the Comprehensive Mental Health Action Plan 2013–2020 in May 2013 [44], establishing as one of its objectives the prioritization of child and adolescent mental health through policies and laws to protect children and adolescents, supporting parents or legal guardians to provide loving care, implementing school-based programs, and improving the quality of community and online environments. WHO (2022) [44] states that school-based social and emotional learning programs are among the most effective advocacy strategies for countries at all income levels. However, Severe Mental Disorders (SMD), such as mood, psychotic and personality disorders, have a negative impact because of the high degree of cognitive, emotional, and behavioral distortion [44], as well as the personal, social, and occupational impairment they entail. Therefore, the design and “preliminary implementation” of the educational program “MotivACTION” is proposed to further promote a positive attitude among young students to enable them to face the challenges of everyday life on a personal, social, and educational level.

Regarding the qualitative results about the perception of schoolchildren on the transfer of what they learnt in “MotivACTION” to their daily life at school, in their relationship with their peers and in their relationship with their family, they express through the written expression in item 10 of the ad-hoc questionnaire, the importance of expressing what they feel in different situations that occur in class, in the relationship with peers, the importance of setting up the “Nutriplate” in the family or how they would like to do physical exercise as a family. These findings are consistent with the previous interventions [21] focusing on environmental change and empowering individuals and communities, with a focus on including the family context, addressing early life determinants, as well as the need to reduce childhood obesity without increasing socio-economic inequalities [45].

Remarkably, that many studies indicate the need to apply educational and pedagogical programs that promote emotional competencies (CE), social competencies (CS), physical competencies (CF) (personal care and physical fitness level), self-concept, interpersonal competencies (CI), and adherence to the Mediterranean diet (ADM) [46,47]. Likewise, a recent qualitative study, supported by the Spanish Ministry of Consumer Affairs, on perceptions of healthy eating practices and lifestyle habits in the adolescent population, shows that adolescents’ perception of a healthy lifestyle is based on the connection between mental health and healthy practices understood as having a varied and controlled diet, doing physical exercise, knowing how to manage stress-causing factors such as social comparison, followed by excessive exams, lack of time to do homework and family demands. With special attention to physical exercise, extracurricular sports activities prevail, since, on the contrary, in their free time and recreational time they tend to choose passive activities, especially case of girls. About the motivations for physical exercise, one of the main factors is the person’s interest (intrinsic motivation) and the promotion of educational initiatives for physical exercise during school hours, such as dynamic playgrounds [48].

### 4.1. Limitations of the Study

Among the limitations of the study is the selection of the sample, which was carried out by accessibility and not in a randomized manner, thus compromising the external validity of the study. Another aspect to consider is the research design, which is a descriptive, cross-sectional pilot study. Quasi-experimental studies with pre- and post-intervention data collection with experimental and control groups are necessary to verify the causal relationships between the variables analyzed. Regarding data collection, other quantitative questionnaires with psychometric properties validated in the population under study (Cronbach’s Alpha or McDonald’s Omega values) should be administered. Future lines of research should have a quasi-experimental and longitudinal approach, pretest, and post-test. It would also be necessary to apply a randomized probabilistic sampling technique.

### 4.2. Future Practical Applications of the MotivACTION Program

Based on these aspects, the educational workshops “MotivACTION, feed your superACTION” emerge as an alternative didactic technique based on the incorporation of education in emotional skills, decision-making for problem-solving, nutritional education, and education in a more active lifestyle inside and outside school hours. In this way, it contributes to promoting pro-social behaviors, healthy nutritional behaviors, and the practice of physical exercise in young people. Therefore, this proposal simultaneously promotes emotional and prosocial behaviors [49] and an increase in daily physical activity among young people [37,50]. Future studies are needed with a longitudinal quasi-experimental design with repeated measures (pretest and posttest), with a non-randomized Control Group (CG) and Experimental Group (EG), analyzed using a quantitative methodology (tests and questionnaires) and qualitative methodology (observational analysis). In addition, the aim is to check other variables such as (a) anthropometry (percentage of body composition and body perimeters), (b) nutritional status variables (food records, adherence to the Mediterranean diet measured with the “Kid-Predimed questionnaire”), (c) real motor competence and perceived motor competence of schoolchildren and adolescents, (d) cognitive variables (assessment of executive functions), (e) perception of satisfaction with their lives (at a personal level, at home and in social relations). However, nutrition education and holistic (physical and mental) healthy lifestyle intervention programs would be effective educational trends, as long as financial support from social policies is available [23].

## 5. Conclusions

The young students had high scores on the items assessing the relevance of the workshop content to their personal lives and socio-educational experiences. As the main preliminary results of the pilot study, the schoolchildren who experienced the “MotivACTION” workshop reported high satisfaction with the educational program. They were able to create a healthy menu with the frog chef. They also felt better and happier at the end of it, and they enjoyed practicing physical activity moving to the rhythm of the music while doing mathematical calculations.

The educational workshop “MotivACTION, feed your superACTION” is presented as an integrative educational strategy to encourage emotional education, nutritional education, and the promotion of active lifestyle habits in children and adolescents.

## Figures and Tables

**Table 1:** Socio-demographic characteristics of participants.

Socio-Demographic Variables	Characteristics	Dates
Sex		34.4% boys; 29% girls
Geography and year enrolled and current situation.	Boys and girls School children from the Community of Madrid Primary and secondary school students, the academic year 2021/2022.	43.4% Primary school students; 56.6% secondary student
Socio-economic level	Medium.	
Level of education of parents or guardians	School graduate and intermediate studies.	33% School graduate; 67% intermediate studies

**Table 2:** Workshops carried out in the education program.

Thematic Lines	Theoretical Background	Aim	Activity Example
1. Initial activities		To explain the “what” and “why” of the program.	The concept of MotivACTION is defined in relation to emotions, nutritional education, and physical exercise.
2. Testing Motivation	SDT	To explore the personal commitment and motivation of the participants	Graphically, participants self-assess the level of c commitment and personal motivation to participate in the workshop. Activity called: “Motivational Commitmentometer”.
3. Emotional education	TEI, TPB	To work intra-interpersonal emotional intelligence	Emotions are defined, and an activity involving graphics, figures, and art frames is proposed to recognize emotions and moods.
4. Emotional literacy	TEI	To facilitate the emotional education of children and adolescence	In groups (6–8 students), they design “Emotional Literacy” taking Dr. Hitzig’s emotional literacy as a reference.
5. Thinking quality	SDT, TEI, TPB	The influence of thinking on behaviors	After observing several images of situations that occur in the school environment (class attention, exams, recess), students verbalize their thoughts about it.
6. Physical health and physical activity	SDT, TPB	Awareness about physical health through physical activity	A physical exercise activity (active break) is carried out in which curricular contents (mathematical calculations, natural science contents, geography contents, bodily systems) are related to an exercise technique, e.g., We have to add 200 squats among all participants, “How many squats will you have to perform in your group?”
7. Nutritional education	FAO, AESAN strategy	To learn healthy eating behaviors	Each group of students creates the “Harvard Plate” or “Nutriplate” with the help of “Chef Frog” (an infographic of a frog dressed as a chef who explains the difference between fast food and healthy food).
8. Closing activities		To design a personalized action plan to promote healthy behaviors	Each group of students creates an action plan (a template is provided) to design three healthy behavior strategies: (1) How to identify an “uncomfortable” emotion; (2) thinking about a “negative” thought about oneself and changing it to a “positive” thought; (3) choose 3 healthy foods to include in the daily menu at home.

Note: SDT (Self-Determination Theory); TPB (Theory Planned Behavior); TEI (Theory Emotional Intelligence); FAO (Food and Agriculture Organization of the United Nations); AESAN (Spanish Agency of Food Safety and Nutrition).

**Table 3:** Questionnaire ad-hoc.

Items 1 to 9	M	SD
1. How useful is it for you to work on your way of thinking, your emotions, and your fitness level?	9.1	0.95
2. Have you learned something?	8.7	1.04
3. How much do you feel that becoming more aware of the importance of your thoughts and emotions has contributed to you?	9.1	1.02
4. Would you recommend your peers to participate in this workshop?	8.8	0.92
5. How much do you value continuing to participate in workshops on “MotivACTION”?	8.5	1.08
6. Overall satisfaction with the workshop	8.76	1.07
7. Rate the activity in terms of format	9.80	1.02
8. Rate the activity in terms of organization	9.89	0.91
9. What is your overall satisfaction with what the person who led the workshop communicated to you?	8.9	0.93

M: media; SD: standard deviation.

**Table 4:** The following are some of the most representative responses concerning different contexts: time in school, at home and in a relationship with a peer.

Context	Responses
Day-to-day life at school	(a) To be more attentive to what the teacher is saying. (b) I would have a better time in class. (c) I would feel better if I could express what I feel in stressful situations.
2. Relationship with your family	(a) Tell my parents when I feel bad. (b) Make the “nutriplate” with my siblings for lunch and dinner. (c) To plan to go out more to the countryside and play in the park with my family.
3. Relationships with your classmates	(a) I would have a better time with my colleagues. (b) It is better not to use insults to say things. (c) I can think of sports activities to do with my friends.

## Data Availability

Data and data collection methods be presented in detail, without obscuring results. No raw data. This manuscript has not been submitted another scientific journal. The original research results are novel and not previously published. The translation complies with MDPI’s translation policy.
